# Non-native speaker pause patterns closely correspond to those of native speakers at different speech rates

**DOI:** 10.1371/journal.pone.0230710

**Published:** 2020-04-03

**Authors:** Theresa Matzinger, Nikolaus Ritt, W. Tecumseh Fitch

**Affiliations:** 1 Department of English, University of Vienna, Vienna, Austria; 2 Department of Cognitive Biology, University of Vienna, Vienna, Austria; Nagoya University, JAPAN

## Abstract

When speaking a foreign language, non-native speakers can typically be readily identified by their accents. But which aspects of the speech signal determine such accents? Speech pauses occur in all languages but may nonetheless vary in different languages with regard to their duration, number or positions in the speech stream, and therefore are one potential contributor to foreign speech production. The aim of this study was therefore to investigate whether non-native speakers pause ‘with a foreign accent’. We recorded native English speakers and non-native speakers of German or Serbo-Croatian with excellent English reading out an English text at three different speech rates, and analyzed their vocal output in terms of number, duration and location of pauses. Overall, all non-native speakers were identified by native raters as having non-native accents, but native and non-native speakers made pauses that were similarly long, and had similar ratios of pause time compared to total speaking time. Furthermore, all speakers changed their pausing behavior similarly at different speech rates. The only clear difference between native and non-native speakers was that the latter made more pauses than the native speakers. Thus, overall, pause patterns contributed little to the acoustic characteristics of speakers’ non-native accents, when reading aloud. Non-native pause patterns might be acquired more easily than other aspects of pronunciation because pauses are perceptually salient and producing pauses is easy. Alternatively, general cognitive processing mechanisms such as attention, planning or memory may constrain pausing behavior, allowing speakers to transfer their native pause patterns to a second language without significant deviation. We conclude that pauses make a relatively minor contribution to the acoustic characteristics of non-native accents.

## Introduction

When speaking a foreign language, most non-native speakers can be readily identified by their accents, which are often distinctive depending on native language. To understand how different accents arise, and also to help second language learners to eliminate them to improve intelligibility, it is essential to know which aspects of spoken language contribute to non-native speech production.

When studying foreign accents, it is crucial to distinguish between accents in production, i.e. atypical measurable acoustic characteristics of the speech signal, and accents in perception, i.e. listeners’ identification of accents as non-native [1–3]. Although these factors are probably related, they are not identical, and the focus of the current study is solely on accents in production.

A crucial factor generating distinct foreign accents is that linguistic phenomena vary between languages, and speakers transfer language-specific features from one language to another (e.g.[4]). These language-typical features can range from pronunciation of individual phonemes (e.g. [5,6]) to suprasegmental prosodic features like intonation patterns (e.g. [7,8]). For example, native speakers of Japanese have difficulty distinguishing the phonemes /l/ and /r/ in foreign languages because Japanese has only one similar phoneme (e.g.[9]). Native German learners of English often substitute /d/ or /s/ for /ð/ or /θ/ (as in *this* or *thing*) because the latter two English sounds are not part of the German phoneme inventory [10]. German speakers also typically use a different pitch range than English, making native German speakers of English sound “bored” to English native speakers, whereas native English speakers often sound “over-excited” to German native speakers [11–13]. Such examples could be readily multiplied.

Certain linguistic features seem to contribute less to foreign speech production than others. These features include linguistic phenomena claimed to be language-universal [14] that are transferred to (or acquired easily in) a second language (L2). Such language-universal phenomena are rare, and have been suggested to mainly concern pragmatic, rather than phonological or grammatical, features [15–18].

The current study investigates whether L1 and L2 speakers differ in their pausing behavior when reading aloud, and hence whether different pausing patterns contribute to a ‘foreign accent’ in speech production. Pauses are particularly interesting candidates to investigate non-native accents for several reasons. Due to the natural need to breathe, pauses occur in all of the world’s languages. Still, not all pauses are determined by physiological needs, but have functions closely related to cognition: pauses help structure speech, determine speech tempo and rhythm, plan upcoming utterances, can add rhetorical emphasis, or structure turn-taking [19–23]. Such roles, reflected for example by the durations of pauses or by the positions of pauses in a stretch of speech, may be completely physiologically determined or may be realized similarly in different languages (e.g. [24–26,27] see S1 Table) and thus either transfer without change, or be easy to acquire, and thus not contribute to non-native speech production.

On the other hand, the different functions of pauses potentially make them subject to cross-linguistic variation (see S2 Table). For example, there is evidence that English speakers pause more frequently than French [28,29] or Turkish speakers [30], but less frequently than Spanish speakers [31]. Also, English speakers’ pauses are shorter than French speakers’ [28,32,but also 33] and Russian speakers’ [34], but longer than Italian speakers’ pauses [33]. Such cross-linguistic differences in pause patterns, if carried over to a foreign language, might contribute to non-native accents in speech production. The main aim of this study is to investigate these possibilities, to determine whether people speaking a non-native language pause with a foreign accent when reading aloud.

Previous studies on pausing behavior in non-native languages mostly concern fluency rather than foreign accent (e.g. [32,34–38]). Although a lack of fluency can contribute to recognizing speakers as non-native, fluency should be distinguished from accents, since proficient second language speakers might be highly fluent, but still possess a clear foreign accent [3,39–41]. Nonetheless, some tentative predictions can be drawn from fluency studies (see S3 Table): speakers tend to make more [32,42–47] and longer [32,46,47] (but also [32,42,43]) pauses when speaking their second language (L2) relative to their first language (L1). However, some reports find associations between speakers’ L1 and L2 pausing behavior. When comparing L2 speakers to L1 speakers of the target language, it seems that highly proficient speakers adhere more closely to native-like pause patterns, whereas less proficient speakers make more and longer pauses than L1 speakers (L1 Korean, L2 English 8,L1 Russian, L2 English 34; see S4 Table). In addition, L1 and L2 pause frequency are correlated: while, overall, speakers make more pauses in their L2 than in their L1, speakers’ L2 pausing behavior can be predicted if their L1 pausing behavior is known [47]. Finally, studies on the perception of fluency and accent can lead to conclusions about how pauses influence accents. For example, acoustic measures of fluency (including pause incidence and duration) have been shown to be predictors of accent ratings [3]. Still, perception of foreign accent was only weakly correlated with acoustic fluency measures, which is why, overall, accentedness and fluency can be regarded as two separate, partially independent concepts [3].

An important methodological issue implies that these previous studies cannot be taken as a clear evidence that pauses contribute to foreign accents in production: an intrinsic lack of stimulus control during free speech. Because pauses serve multiple functions, they can be influenced by many different variables, such as speech genre, cognitive load, or syntactic complexity of the utterances [34,48]. These diverse influencing factors make it a challenge to tease out the contributions of pauses to foreign accents in spontaneous speech (e.g., picture description tasks or spontaneous monologues). Differences in pause patterns may arise not due to foreign transference, but due to different sentence structures employed or speech styles adopted by the speakers, which cannot be controlled in spontaneous speech. Additional factors such as communicative intent, personal speaking style, or emotional involvement with the speech task [34,47–49] are equally difficult to control for. Most previous studies on second language pausing controlled for some of these potential factors but, due to their focus on second language fluency, did not control enough factors to reliably attribute foreign accents to pause differences.

Here we introduce an experimental procedure which integrates and modifies methods from previous studies, safeguarding against potential confounds, to focus on the specific contribution of pauses to foreign accents in speech production. We compared the pausing behavior of L2 speakers of English to the pausing behavior of L1 speakers of English, reading out the same scripted text at different speech rates. We measured multiple characteristics of pauses: pause-to-utterance ratio (total pause time in relation to total speaking time), the mean duration of pauses, the number of pauses that speakers made, and the positions in the written text at which they occurred.

By having all speakers read the same written text, we could exclude the influence of morpho-syntactic factors (e.g. word length, word structure, and syntactic structure) that might underlie previous findings of language differences in pausing. Also, we reasoned that the cognitive load involved in reading a text is reduced compared to free speech. Thus, by using written text as a prompt, we aimed to limit the occurrence of vocal hesitations resulting from a lack of L2 fluency to distinguish fluency from foreign accent per se.

To examine whether unusual reading conditions affect pausing behavior, we investigated speakers’ pausing performance at three different reading speeds (casual, slow and fast speech rate), aiming to disentangle the role of cognitive load on the realization of pauses. We reasoned that the cognitive load should be lowest in casual reading speed because speakers encounter this speech tempo frequently in daily life and therefore have more possibilities to acquire native-like pause patterns [50]. Accents might preferentially surface in unnatural reading conditions, particularly in speeded reading where the cognitive load is higher and speakers might fall back on cognitive mechanisms developed for their native language. Also, testing the same individuals at different reading speeds offers a within-individual manipulation, helping to address individual differences in speaking and/or pausing styles.

We tested non-native speakers with two different L1 backgrounds, namely German or Serbo-Croatian L1. Typologically, both English and German belong to the Germanic language family, and are stress-based languages [51], whereas Serbo-Croatian is a Slavic language [52] and is not stress-based [25]. This selection of L1 speakers thus would allow comparison of pausing by English native speakers with those of L2 speakers with an L1 background more (German) or less (Serbo-Croatian) similar to English. However, this comparison was not included in the final analysis due to a low sample size of Serbo-Croatian speakers.

Summarizing, we used a standardized procedure to compare pause patterns (pause-to-utterance ratio, pause duration, pause number, pause positions) of native speakers of English and two non-native English speaker groups reading out the same text at three different speech rates. Previous work leads to several hypotheses and predictions.

According to the *No Contribution* hypothesis, pauses do not contribute to non-native speech production. This is predicted if the acquisition of pause patterns is simple during language acquisition, because pauses are perceptually highly salient (Matzinger, Ritt, Fitch, in prep) and pausing is articulatorily simple (compared to the articulation of other vocal elements like vowels, consonants, or intonation). Alternatively, L2-typical pause patterns might result from similar pause patterns in the speakers’ L1 being transferred to their L2, or even reflect language-universal pausing behavior. By the *No Contribution* hypothesis, accents are caused by non-native realizations of linguistic features other than pauses. These non-native realizations might for example concern phonemes, word stress patterns or prosody [2,39]. Besides that, non-nativeness might be signaled by atypical gestures or turn-taking behavior. The *No Contribution* hypothesis predicts no differences in the pausing behavior of native and non-native speakers of English: speakers should pause at the same syntactic positions, equally often and for similar durations as native speakers. If pauses can be acquired easily or are language-independent, this hypothesis also predicts no interactions between nativeness and reading speed: L2 speakers should perform similarly to L1 speakers whether reading fast or slowly.

Alternatively, the *Pause Contribution* hypothesis postulates that pauses contribute to foreign accents. This might be due to a higher cognitive load (e.g. processing or memory constraints [18,53]) when speaking an L2 [8,54,55]. Alternatively, differences in the typical pausing behavior of speakers’ L1 and L2 may make the typical pause characteristics of the L2 difficult to attain, because speakers’ native pausing pattern overrides the learned non-native pattern. The *Pause Contribution* hypothesis predicts that pause patterns of L2 speakers will differ significantly from pause patterns of L1 speakers. If differences result from a higher cognitive load when speaking the L2, there should be more and longer pauses in L2 speakers than in L1 speakers. Also, this predicts an interaction between nativeness and reading speed. Differences between L1 and L2 pausing should be smaller in casual reading speed than in unnatural reading conditions (i.e. fast or slow speech), which pose a higher cognitive load because they are encountered and practiced less frequently. L2 speakers might therefore have had fewer chances to acquire them in a native-like manner and might fall back on non-native patterns instead. Differences are predicted to be higher in fast than in slow reading aloud, because reading rapidly should be more cognitively challenging than reading slowly.

## Materials and methods

### Target languages and participants

We obtained speech samples from 41 participants of three different first languages: English native speakers (13 participants; 7f; mean age: 35.2) and non-native English speakers with German (18 participants; 10f; mean age: 29.5) and Serbo-Croatian (10 participants; 6f; mean age: 25.9) as their first languages. Participants were university students or staff recruited individually at the University of Vienna. All non-native English participants were advanced learners of English, who did not have diagnosed reading or speaking difficulties, self-assessed themselves as being proficient in English (equivalent to CEFR level C1) and reported in post-experiment questionnaires (see S1 Appendix) to be concerned with English regularly both in the productive and receptive domain (e.g. in the university or work context, media exposure).

Nonetheless, and crucially, in a native language recognition test with our pool of speech samples, five English native speakers (m, mean age: 41.2) could still detect all non-native speakers due to their distinctive accents. This native language recognition test ensured that our non-native speakers qualified for the study: being identified as non-native in the accent recognition test suggests that certain features of native and non-native speech production differ. These might potentially include deviations in pause patterns.

The language recognition test was implemented using the software package PRAAT (Version 6.0.36, [56]). Raters listened to a speech sample of each participant reading out the target text (see below) in casual speech tempo. The task of the raters was to indicate if they believed the speakers to be native speakers of English, German or Serbo-Croatian. The raters controlled the timing, moving to the next speech sample as soon as they were sufficiently certain about their decision.

Although four German native speakers were misclassified as English native speakers by one or two of the raters each, the other raters correctly identified them as non-native. Also, one Serbo-Croatian native speaker was misclassified as an English native speaker by one of the raters, but correctly recognized as non-native by all other raters. The five raters correctly recognized all English native speakers, except that four English native speakers were classified as German L2 speakers by one rater each, and one of these English native speakers was additionally classified as a Serbo-Croatian L2 speaker by a second rater (see S5 Table). We concluded from these ratings that our speakers qualified for the analysis.

The study protocol was approved by the ethics board of the University of Vienna (reference number: #00333/00384). All subjects gave written informed consent in accordance with the Declaration of Helsinki.

### Speech recordings

Speech samples were collected by recording participants reading out the English prose text *The boy who cried wolf* (see S2 Appendix), a fable frequently used for evaluating English pronunciation [57]. The recordings were made with a ZOOM Handy Recorder (H4n, ZOOM Corporation, Japan) either in a sound-proof room or in a quiet office environment. We recorded the participants reading out the text in three different speech tempi: fast, casually and slowly. Participants were instructed to read the text casually in the *casual* condition, to read as fast as they could in the *fast* condition and to read the text slowly (e.g., as if to a group of preschool children), in the *slow* condition. The order of the different tempo conditions was randomized for each participant. To elicit a natural reading style and minimize pauses resulting from hesitation due to unfamiliarity, participants were asked to read the text silently before recording in order to familiarize themselves with the text to avoid them being distracted by unfamiliar words or content during recording. In addition, before the actual recording, participants read the first sentence of the text aloud to make them comfortable reading aloud in the experimental setting, while the experimenter adjusted the signal recording levels.

### Measurements and analyses

For determining pauses in the recordings of participants reading aloud, a pause was defined as a period of silence with a minimal duration of 0.1 seconds, most likely occurring for breathing, rhythmic or pragmatic reasons (the choice of this threshold is explained in more detail in Box 1).

**Box 1. What is a pause?**

Previous studies differ considerably in what they consider the lower durational threshold for classifying silent intervals in speech recordings as pauses (reviewed in [37,80]). These threshold values start as low as 5 ms (e.g. [25]) and range up to values as high as 400 ms [45,53,81]. 200 ms is a popular threshold for pauses in L2 speech [37,80]. The choice of a particular lower durational threshold is often determined by the type of pauses investigated in a particular study. For example, studies concerned with pauses resulting from a lack of fluency in an L2 tend to choose longer durational thresholds than studies investigating pauses in L1 everyday conversation. For our purpose, it was essential to sample all pauses, without excluding pauses below a lower durational limit. Still, we could not automatically classify all silent intervals in our recordings as pauses because silent intervals can be of multiple origins.

Silent intervals in speech recordings can occur because of pauses that fit the definition for our analysis, i.e. silent intervals resulting from breathing, rhythmic, structural or pragmatic reasons (“true pauses”), but also because of holds in stop consonants (for example /p/, /t/ or /k/) or very low amplitude schwas or fricatives (for example /f/, /s/ or /h/), i.e. silent intervals that are not considered as pauses (“phonetic silences”). Our automatic pause detection algorithm should only detect the former pauses, but not the latter ones. In order to determine the lower durational limit for the automatic detection mechanism, we determined the threshold below which no more true pauses occurred. For that, we analyzed the recordings of 6 speakers (pseudo-randomized, we ensured that there were 2 speakers of each language, one male and one female, 2 recordings for each condition, and 3 speakers in the sound-proof room and in the office). We automatically detected all silent intervals longer than 0.001 seconds (threshold -35 dB, minimum silent interval: 0.001 s) and then manually determined whether the detected silences were phonetic silences or true pauses. We then evaluated the distributional pattern of silences.

**Fig 1:**
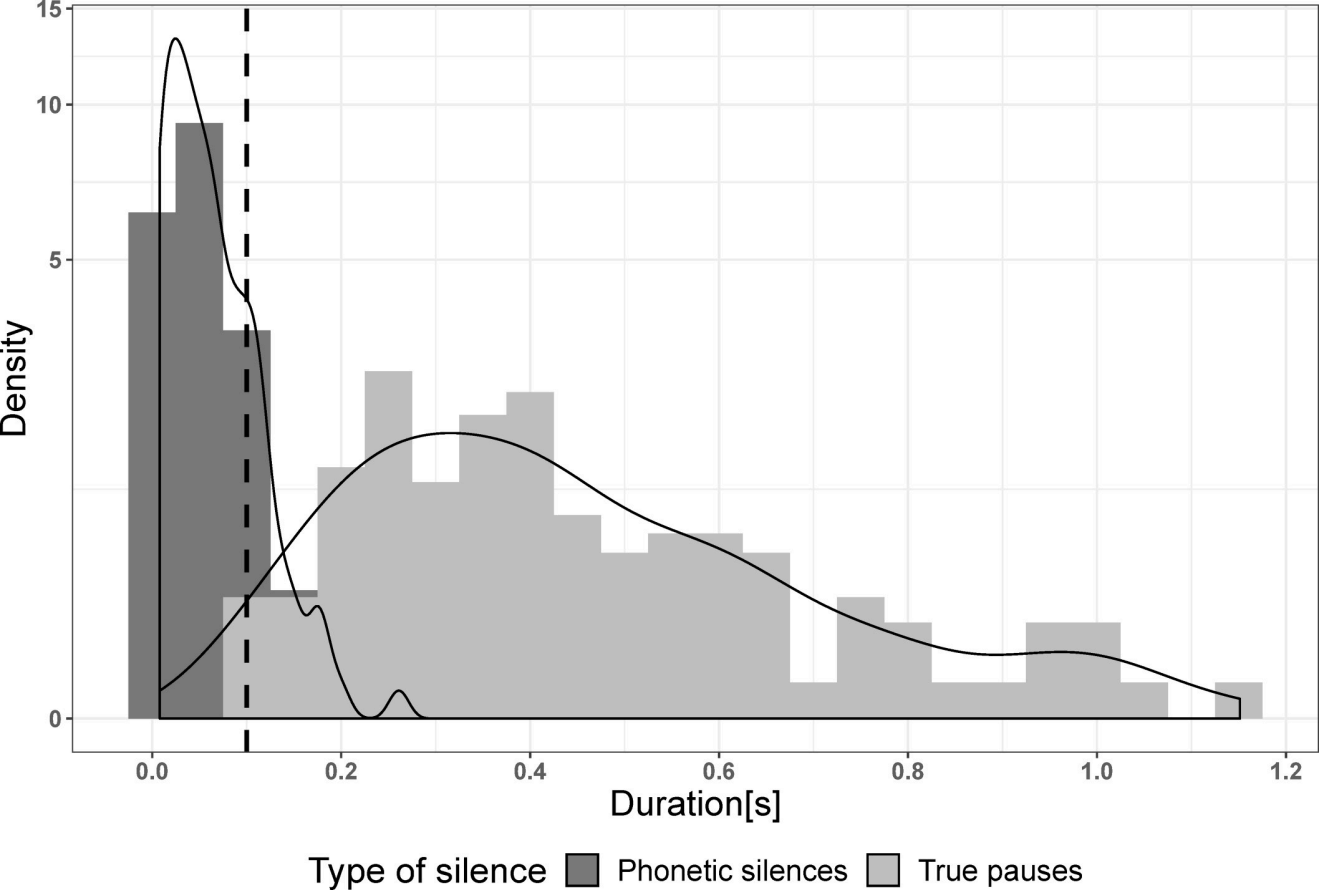
Distribution of phonetic silences and true pauses. Dashed line = threshold chosen for the subsequent automatic detection of pauses (0.1 s).

In these analyses we found that 90.39% (CIs: 80.89 and 99.9%) of the phonetic silences (n = 338) were shorter than 0.1 s, 9.29% (CIs: 0.44 and 18.14%) had durations between 0.1 and 0.2 s, and 0.32% (CIs: -0.5 and 1.13%) were longer than 0.2 s. In contrast, 93.14% (CIs: 86.09 and 100.1%) of the pauses (n = 132) were longer than 0.2 s, 6.86% (CIs: -0.18 and 13.91%) had durations between 0.1 and 0.2 s, and no true pauses were shorter than 0.1 s (Fig 1, Table 1). This led us to the conclusion to choose 0.1 s as a lower threshold for the automatic annotation of pauses to exclude most phonetic silences and include all true pauses. The remaining phonetic silences were deleted manually.

**Table 1:** Mean pause duration and mean proportion of short (< 0.1 s), medium (0.1 < x < 0.2 s) and long (> 0.2 s) phonetic silences and true pauses with respective low and high confidence intervals (CIs).

	Duration [s]	Low CI	High CI	Short [%x]	Low CI	High CI	Medium [%]	Low CI	High CI	Long [%]	Low CI	High CI
Phonetic silences	0.047	0.033	0.061	90.39	80.89	99.9	9.29	0.44	18.14	0.32	-0.5	1.13
True pauses	0.404	0.301	0.506	0	0	0	6.86	-0.18	13.91	93.14	86.09	100.18

Pause measurement was performed using the software package PRAAT (Version 6.0.36, [56]). For that purpose, pauses were automatically annotated (Annotate → To TextGrid (silences); guidelines for settings: Silence threshold: -35.0 dB, Minimum silent interval duration: 0.1 s, Minimum sounding interval duration: 0.1 s). Additionally, all pauses were checked visually (in the oscillogram and spectrogram) and acoustically, and adjusted manually, in order to remove rarely occurring incorrectly identified pauses (e.g. holds in plosives, low amplitude fricatives; see Box 1) or to insert pauses that had not been automatically detected (for example, because of breathing noise). With a PRAAT script, the total duration of each reading, the number of pauses and the duration of individual pauses in each reading were extracted. We included all true silent pauses (see Box 1). Although we intended to include filled pauses that contained a noise component resulting for example from breathing or from vocal hesitations such as “ehm” or “uh” [21,48,58,59], we did not find vocal hesitations in our data, most likely because participants read a scripted text and had familiarized themselves with the text before being recorded. Thus, the only filled pauses in our data are rarely occurring intervals containing obvious breathing noise.

We classified all pauses with regard to their position in the text. For each pause, we determined whether it occurred at a punctuation mark in the text (hereafter “marked pauses”; i.e. full stops, commas or quotation marks; no other punctuation marks occurred in the text), at an unmarked clause or phrase boundary (hereafter “unmarked pauses”; e.g. before a defining relative clause), or at any other position in the text. For the full text with the annotation of the pause categories see S2 Appendix.

We used linear and logistic mixed effects models to investigate the influence of reading tempo, native language and in-text position on the realization of pauses. Preliminary analyses did not reveal differences between native German and native Serbo-Croatian non-native speakers of English, so we lumped these two groups together for our analyses. Thus our analyses compared two groups, namely native and non-native speakers of English (“nativeness” factor). Still, in our plots, we present the data for all three native languages separately, in order to allow visual comparisons between them.

For our model predictors reading tempo and nativeness, we used deviation coding [60]. Reading tempo was coded as a continuous predictor (fast = -0.5, casual = 0, slow = +0.5), and nativeness was coded as a two-level factor (native = -0.5, non-native = +0.5).

To test whether total reading time, pause-to-utterance ratio and the duration of individual pauses were influenced by reading tempo and nativeness, we used linear mixed models [61] into which we entered these two predictors and an interaction term of the two as fixed effects. In order to reduce non-normality in the error structure of our models, the dependent variables total reading time and duration of individual pauses were log-transformed, because the optimal lambda for a Box-Cox transformation [62] was close to 0 in both cases, using the *boxcox* function of the *MASS* package [63]. The dependent variable pause-to-utterance ratio, which is proportional data bounded by 0 and 1, was logit-transformed, using the *logit* function of the *boot* package [64–67].

To investigate which factors influenced the probability of making a pause, we used a generalized linear mixed model [61] with binomial error structure and a logit link function. Each transition between two words represented a data point, and we determined for each of these transitions if a pause occurred there or not (similar to [38]). In total, this resulted in 26,456 data points. This number of data points can be explained as follows: 41 participants * 3 reading tempi * 215 word boundaries in the text = 26,445 data points. Additional 11 datapoints resulted from words that were not in the scripted text but that participants inserted spontaneously while reading. This yielded 26,456 data points in total, 2,750 of which were pauses. In this model, we included reading tempo, nativeness, in-text position and an interaction of reading tempo and nativeness as fixed effects.

All of our models included participant as a random intercept. For the three models testing the influence of reading tempo and nativeness on total reading time, pause-to-utterance ratio and the duration of individual pauses, our design, with one reading per tempo condition of each participant, did not allow us to accurately estimate random slopes. For the model testing the influence of reading tempo, nativeness and in-text position on the probability of making a pause, we ran an initial model that also included a random slope of position. However, this model did not converge. Thus, no random slopes are included in the models (but see [68–70]).

All models were fitted in R (version 3.5.1, [71]) and implemented in RStudio (version 1.1.456, [72]) using the *lmer* function of the *lme4* package [73].

For each linear mixed model, we visually inspected a qqplot and the residuals plotted against fitted values to check whether the assumptions of normally distributed and homogeneous residuals were fulfilled (using a function provided by Roger Mundry, Leipzig, Germany). These indicated no obvious deviations from normality or homoscedasticity.

Finally, we derived variance inflation factors (VIF, [74]) using the *vif* function of the R-packagae *car* applied to our models with the random effects excluded. They did not indicate collinearity to be an issue. We tested the significance of the respective full models as compared to the null models (comprising only the random intercept) by using a likelihood ratio test (R function *anova* with the argument test set to “Chisq”, [75,76]). In all cases, parameters were estimated using maximum likelihood (rather than Restricted Maximum Likelihood, [77]) in order to allow for likelihood ratio tests. To obtain p-values for the individual effects, we conducted likelihood ratio tests comparing the full with respective reduced models ([69], R function *drop1*).

As indicators for the goodness-of-fit of our models, we follow [78] and report the marginal and conditional R^2^ for each full model. The marginal R^2^ (R^2^_m_) reveals the variance explained by the entirety of the fixed effects, and the conditional R^2^ (R^2^_c_) reveals the variance explained by the entirety of the fixed and random effects. Thus, these measures can be taken as indicators for the effect size for the full models. We calculated R^2^_m_ and R^2^_c_ using the *r*.*squaredGLMM* function from the *MuMIn* package [79].

## Results

**Fig 2:**
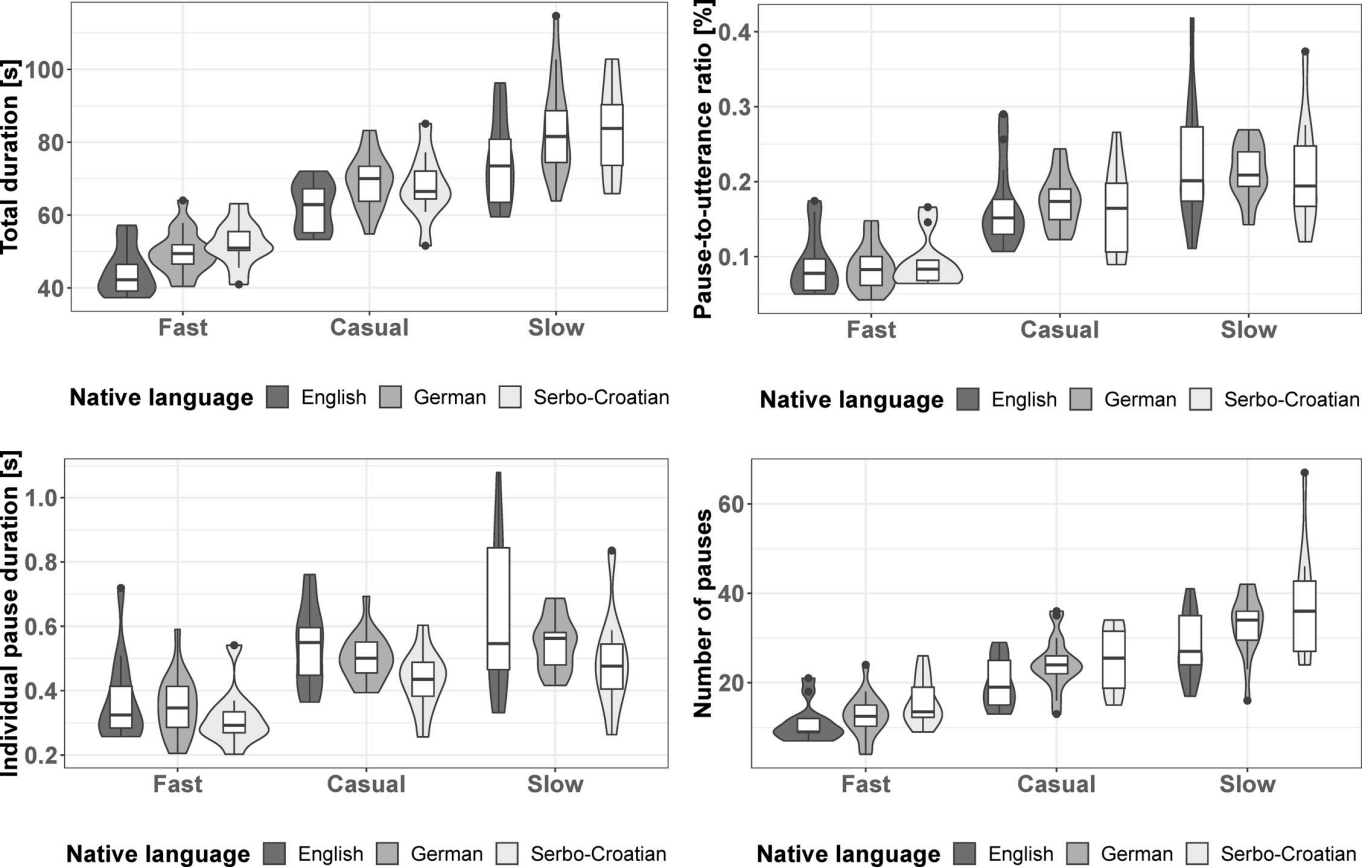
**a)** total duration of the readings in seconds, **b)** pause-to-utterance ratio in %, **c)** duration of individual pauses in seconds and **d)** number of pauses in each condition for each native language. The violin plots show median values (horizontal black lines) with first and third quartiles (lower and upper end of boxes), minimum and maximum values limited to values no more than 1.5 IQRs distant from the respective end of the box (lower and upper end of vertical black lines) and outliers (black dots). The area around each box indicates the distribution of the data.

Our instructions successfully elicited three desired reading aloud tempi: the full model for the total reading time was clearly significant compared to the null model (likelihood ratio test: χ^2^ = 169.43, df = 3, p < 0.001, effect size for the full model: R^2^_m_ = 0.72, R^2^_c_ = 0.82). Specifically, there was an effect of reading tempo on total reading time (likelihood ratio test: χ^2^ = 163.67, df = 1, p < 0.001), with reading time increasing from the fast to the slow condition. Furthermore, we found a significant main effect of nativeness on the total reading time (likelihood ratio test: χ^2^ = 10.21, df = 1, p = 0.001) with the total reading duration being higher in non-native speakers than in native speakers of English. There was no significant interaction effect of reading tempo and nativeness, i.e. non-native speakers did not change their reading durations differently in fast and slow tempo compared to native speakers (Table 2; Fig 2A; random effects: S8 Table).

**Table 2:** Results of the linear mixed model exploring the effects of reading tempo and nativeness on the total reading time (log-transformed). The table reports estimated model coefficients, standard errors (SE) and lower and upper confidence intervals (CI), χ^2^ values of likelihood ratio tests and respective degrees of freedom (df) and p-values (P).

Term	Estimate	SE	lower CI	upper CI	χ^2^	df	P
**Intercept**	4.07	0.03	4.02	4.13	^a^	^a^	^a^
**Reading tempo**^b^	0.51	0.04	0.43	0.60	163.67	1	< 0.001
**NativenessNonNative**^b^	0.11	0.03	0.05	0.18	10.21	1	0.001
**Reading tempo:NativenessNonNative**^**(2)**^	-0.02	0.05	-0.18	0.08	0.11	1	0.740

^a^ not shown because of having a very limited interpretation

^b^ Reading tempo and nativeness were deviation coded: reading tempo was coded as a continuous predictor (fast = -0.5, casual = 0, slow = +0.5), and nativeness was coded as a two-level factor (native = -0.5, non-native = +0.5); the indicated tests were obtained from likelihood ratio tests comparing the full with a reduced model lacking reading tempo, nativeness and the interaction, respectively.

We next explored how reading tempo and nativeness influenced the realization of pauses. In particular, we investigated the proportion of the total reading time that was devoted to pauses (pause-to-utterance ratio; Fig 2B), and how long the individual pauses were (individual pause duration; Fig 2C) at the different reading tempi in native and non-native speakers. We also explored how many pauses speakers made (number of pauses; Fig 2D) and how the probabilities that speakers made pauses at certain positions in the text were influenced by reading tempo and nativeness.

### The effects of reading tempo and nativeness on the pause-to-utterance ratio

The full model for the pause-to-utterance ratio was significant compared to the null model (likelihood ratio test: χ^2^ = 138.08, df = 3, p < 0.001, effect size for the full model: R^2^_m_ = 0.58, R^2^_c_ = 0.80). More specifically, reading tempo had a significant effect on the pause-to-utterance ratio (likelihood ratio test: χ^2^ = 137.94, df = 1, p < 0.001). In contrast, we did not find a significant effect of nativeness on the pause-to-utterance ratio (likelihood ratio test: χ^2^ = 0.002, df = 1, p = 0.965). Also, the effect of the interaction of reading tempo and nativeness was non-significant (likelihood ratio test: χ^2^ = 0.14, df = 1, p = 0.709). Non-native speakers thus spent a similar amount of time on pauses as native speakers.

Averaged over native and non-native speakers, the mean pause-to-utterance ratio was 22.62% for slow, 14.35% for casual, and 8.76% for fast reading aloud (Table 3; Fig 2B; random effects: S9 Table). We used the following formulae for the back-transformation from the logit-transformed model estimates (Table 3): odds = exp(– 1.79 + 1.11 * reading tempo + 0.01 * nativeness– 0.05 * reading tempo * nativeness, and y = odds/(1+odds). Reading tempo and nativeness were deviation coded: reading tempo was coded as a continuous predictor (fast = -0.5, casual = 0, slow = +0.5), and nativeness was coded as a two-level factor (native = -0.5, non-native = +0.5). The respective values for fast, casual and slow reading speeds were inserted into the formulae to calculate the mean pause-to-utterance ratios. To get values averaged for native and non-native speakers, we inserted 0 for nativeness.

**Table 3:** Results of the linear mixed model exploring the effects of reading tempo and native language on pause-to-utterance ratio (logit-transformed). The table reports estimated model coefficients, standard errors (SE) and lower and upper confidence intervals (CI), χ^2^ values of likelihood ratio tests and respective degrees of freedom (df) and p-values (P).

Term	Estimate	SE	lower CI	upper CI	χ^2^	df	P
**Intercept**	-1.79	0.09	-1.96	-1.61	^a^	^a^	^a^
**Reading tempo**^b^	1.11	0.10	0.91	1.32	137.94	1	< 0.001
**NativenessNonNative**^b^	0.01	0.10	-0.20	0.21	0.002	1	0.965
**Reading tempo: NativenessNonNative**^**(2)**^	-0.05	0.12	-0.29	0.20	0.14	1	0.709

^a^ not shown because of having a very limited interpretation

^b^ Reading tempo and nativeness were deviation coded: reading tempo was coded as a continuous predictor (fast = -0.5, casual = 0, slow = +0.5), and nativeness was coded as a two-level factor (native = -0.5, non-native = +0.5); the indicated tests were obtained from likelihood ratio tests comparing the full with a reduced model lacking reading tempo, nativeness and the interaction, respectively.

### The effects of reading tempo and nativeness on the duration of individual pauses

The full model for the individual pause durations was significant compared to the null model (likelihood ratio test: χ^2^ = 89.46, df = 3, p < 0.001, effect size for the full model: R^2^_m_ = 0.37, R^2^_c_ = 0.73). We found that there was a significant effect of reading tempo on the duration of individual pauses (likelihood ratio test: χ^2^ = 87.53, df = 1, p < 0.001). Contrastingly, we did not find a significant effect of nativeness on pause duration (likelihood ratio test: χ^2^ = 1.73, df = 1, p = 0.188). Likewise, the effect of the interaction of reading tempo and nativeness was non-significant (likelihood ratio test: χ^2^ = 0.21, df = 1, p = 0.651), which indicates that native and non-native speakers of English altered the duration of their pauses similarly in different reading tempi.

The mean duration of individual pauses, averaged over native and non-native speakers, was 0.60 s in slow, 0.47 s in casual, and 0.37 s in fast reading aloud (Table 4; Fig 2C; random effects: S8 Table). We used the following formula for the back-transformation from the log-transformed model estimates (Table 4): y = exp(– 0.75 + 0.49 * reading tempo– 0.10 * nativeness– 0.04 * reading tempo * nativeness). Reading tempo and nativeness were deviation coded: reading tempo was coded as a continuous predictor (fast = -0.5, casual = 0, slow = +0.5), and nativeness was coded as a two-level factor (native = -0.5, non-native = +0.5). The respective values for fast, casual and slow speech were inserted into the formula to calculate the mean durations. To get values averaged for native and non-native speakers, we inserted 0 for nativeness.

**Table 4:** Results of the linear mixed model exploring the effects of reading tempo and native language on the duration of individual pauses (log-transformed). The table reports estimated model coefficients, standard errors (SE) and lower and upper confidence intervals (CI), χ^2^ values of likelihood ratio tests and respective degrees of freedom (df) and p-values (P).

Term	Estimate	SE	lower CI	upper CI	χ^2^	df	P
**Intercept**	-0.75	0.06	-0.87	-0.63	^a^	^a^	^a^
**Reading tempo**^b^	0.49	0.07	0.36	0.62	87.53	1	< 0.001
**NativenessNonNative**^b^	-0.10	0.07	-0.24	0.05	1.73	1	0.188
**Reading tempo: NativenessNonNative**^**(2)**^	-0.04	0.08	-0.19	0.12	0.25	1	0.651

^a^ not shown because of having a very limited interpretation

^b^ Reading tempo and nativeness were deviation coded: reading tempo was coded as a continuous predictor (fast = -0.5, casual = 0, slow = +0.5), and nativeness was coded as a two-level factor (native = -0.5, non-native = +0.5); the indicated tests were obtained from likelihood ratio tests comparing the full with a reduced model lacking reading tempo, nativeness and the interaction, respectively.

### The effect of reading tempo, nativeness and position in the text on the occurrence frequency of pauses

The full model for the occurrence frequency of pauses was significant compared to the null model (likelihood ratio test: χ^2^ = 10186, df = 5, p < 0.001, effect size for the full model: R^2^_m_ = 0.32, R^2^_c_ = 0.34). We found significant effects of reading tempo (likelihood ratio test: χ^2^ = 906.75, df = 1, p < 0.001), of nativeness (likelihood ratio test: χ^2^ = 7.05, df = 1, p = 0.008) and of in-text position (likelihood ratio test: χ^2^ = 9759.82, df = 1, p < 0.001) on the occurrence frequency of pauses. Non-native speakers made more pauses than native speakers, and people made more pauses the more slowly they read aloud. Also, people made more pauses at punctuation marks than at unmarked phrase boundaries and at other positions in the text. However, the effect of the interaction of reading tempo and nativeness was non-significant (likelihood ratio test: χ^2^ = 2.59, df = 1, p = 0.11), which indicates that native and non-native speakers of English altered the occurrence frequency of their pauses similarly in different reading aloud tempi (Table 5). Regarding our random intercept of participant, the estimated standard deviation among participants was 0.56 (S9 Table). This is smaller than the magnitude of effects of in-text position and reading tempo, but similar to the magnitude of the effect of nativeness (cf. Table 5). This indicates that the influence of participant variability and nativeness are comparable.

**Table 5:** Results of the logistic regression model exploring the effects of reading tempo, native language and in-text position on the occurrence frequency of pauses. The table reports estimated model coefficients, standard errors (SE) and lower and upper confidence intervals (CI), χ^2^ values of likelihood ratio tests and respective degrees of freedom (df) and p-values (P).

Term	Estimate	SE	lower CI	upper CI	χ^2^	df	P
**Intercept**	0.60	0.17	0.27	0.94	^a^	^a^	^a^
**PositionUnmarkedPhraseBoundary**^b^	-2.73	0.08	-2.89	-2.57	9759.82	1	< 0.001^c^
**PositionOtherWordBoundary**^b^	-6.06	0.09	-6.25	-5.88			
**Reading tempo**^b^	2.20	0.15	1.91	2.48	906.75	1	< 0.001
**NativenessNonNative**^b^	0.54	0.20	0.14	0.94	7.05	1	0.008
**Reading tempo: NativenessNonNative**^b^	0.27	0.17	-0.06	0.60	2.59	1	0.11

^a^ not shown because of having a very limited interpretation

^b^ In-text position was dummy coded with the marked position being the respective reference category. Reading tempo and nativeness were deviation coded: reading tempo was coded as a continuous predictor (fast = -0.5, casual = 0, slow = +0.5), and nativeness was coded as a two-level factor (native = -0.5, non-native = +0.5); the indicated tests were obtained from likelihood ratio tests comparing the full with a reduced model lacking in-text position, reading tempo, nativeness and the interaction between reading tempo and nativeness, respectively.

^c^ This is the overall effect of in-text position on the occurrence frequency of pauses. Considering the differences between the individual levels, the model revealed that at unmarked phrase boundaries (z = -33.49, p < 0.001) and at other word boundaries (z = -65.17, p < 0.001), participants paused significantly less than at punctuation marks.

**Fig 3:**
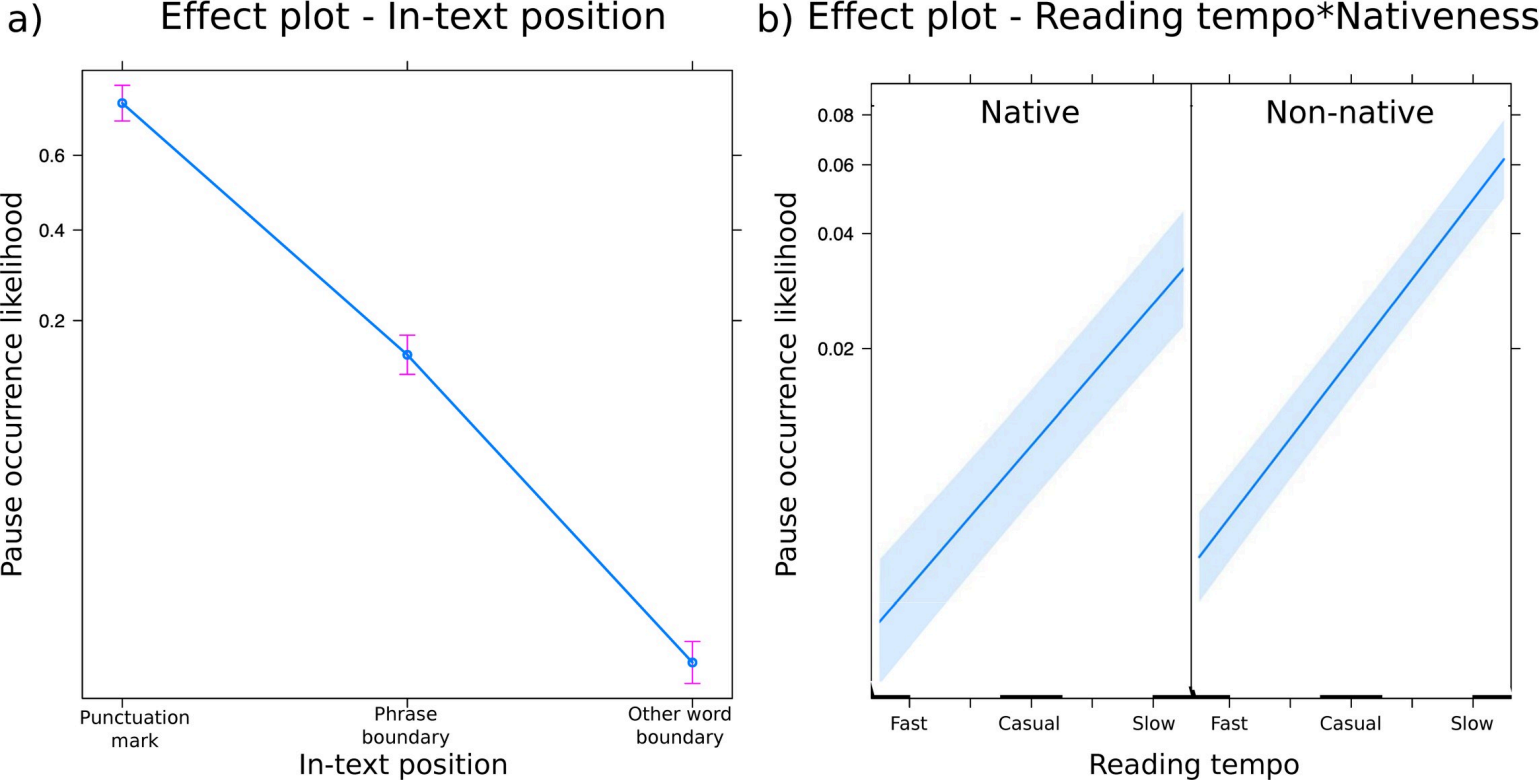
**a)** effect display of the significant main effect of in-text position, **b)** effect display of the significant main effects of reading tempo and nativeness. The effect of the interaction of reading tempo and nativeness was non-significant. The y-axis displays the probability of occurrence of a word after a pause. Error bars and shaded areas around the estimated effects represent 95% confidence intervals.

Native and non-native speakers’ predicted probabilities of making a pause in fast, casual and slow reading at punctuation marks, unmarked phrase boundaries and other word boundaries are given in Table 6. The corresponding partial effects of our model are shown in Fig 3. The overall numbers of pauses in each reading tempo and each native language are displayed in Fig 2D.

**Table 6:** Predicted probabilities (in %) of making a pause in fast, casual or slow reading at punctuation marks, unmarked phrase boundaries or other positions in the text for native and non-native speakers of English.

	Native^a^			Non-Native^a^		
	**Fast**	**Casual**	**Slow**	**Fast**	**Casual**	**Slow**
**Punctuation mark**	33.20	58.19	79.58	42.69	70.50	88.46
**Phrase boundary**	3.15	8.34	20.31	4.65	13.51	33.39
**Other word boundary**	0.12	0.32	0.90	0.17	0.55	1.76

^a^Values are derived from the estimates of our logistic regression model (see Table 5). We used the following formulae for the back-transformation from the logit-transformed model estimates: odds = exp(0.60–2.73 * unmarked phrase boundary– 6.06 * other word boundary + 2.20 * reading tempo + 0.54 * nativeness + 0.27 * reading tempo * nativeness, and y = odds/(1+odds). In-text position was dummy coded, with marked boundaries serving as the reference category. Reading tempo and nativeness were deviation coded: reading tempo was coded as a continuous predictor (fast = -0.5, casual = 0, slow = +0.5), and nativeness was coded as a two-level factor (native = -0.5, non-native = +0.5). The respective values were inserted into the formulae to calculate the predicted probabilities of pause occurrence.

## Discussion

Our study evaluated whether pauses contributed to the speakers’ non-native L2 production by examining whether native and non-native speakers of English differed in their pausing behavior when reading aloud at different speech rates. Although there were some rare misclassifications in our native language recognition test, overall, all non-native speakers were identified as non-native by native English raters, and thus had clearly recognizable non-native accents. However, our findings suggest that non-native pause patterns contributed little to the production of these non-native accents, at least for our relatively proficient speakers (see below). This supports the *No Contribution* hypothesis.

First, native and non-native speakers had similar pause-to-utterance ratios. Second, the durations of their pauses were similar (in line with 25,33,but in contrast to 46). Third, for native and non-native speakers, reading tempo had a similar significant influence on all of the pause characteristics measured, and there were no interactions between nativeness and reading rate. With an increasing reading tempo, native and non-native speakers made fewer and shorter pauses, and with a decreasing reading tempo, they made more and longer pauses. Thus, with changing reading tempo, native and non-native speakers altered both the duration and the occurrence likelihood of pauses in a highly similar way. During fast reading, native and non-native speakers’ probabilities of making a pause were below 5% at unmarked phrase boundaries and below 0.2% at other unmarked positions in the text. Thus, almost only pauses at punctuation marks remained, suggesting that the visually salient punctuation marks help readers to structure their vocal output in similar ways.

Pause-to-utterance ratio changed with changing reading tempo (cf. similar findings in speakers of Dutch, French and Italian; [26]), indicating that in fast reading, pauses were reduced more compared to casual reading and in slow reading, pauses increased relative to casual reading. A potential explanation is that when reducing pauses, there is less information loss compared to reducing articulated sounds. If articulated sounds were deleted or shortened too much, words would be distorted to intelligibility and semantic content would be lost. Similarly, if segments were lengthened to an unnatural extent they would be difficult to produce and perceive. These factors apply much less to shortened or lengthened pauses, and reducing or increasing pause length is articulatorily simpler than varying the duration of vocal segments. This may explain the similar results regarding reading tempo for native and non-native speakers.

The only significant difference in pauses that may contribute to sounding foreign is the higher likelihood of having a pause in L2 speech: non-native speakers made more pauses than native speakers (in contrast to [25]). Although our non-native participants were highly proficient in English, they might still need more time for cognitive processing when speaking their L2 [46,82]. This might be reflected in their higher likelihood of occurrence of pauses (but interestingly not in longer durations of pauses). However, the reason speakers need more processing time for the foreign language might be rooted not in difficulties in adhering to target language pause patterns, but to other aspects of the L2, such as difficulties pronouncing L2-typical sounds. Further evidence that speakers need more time for cognitive processing in their L2 than in their L1 is that non-native speakers had longer reading durations than native speakers (cf. [83]). To rule out the possibility that non-native speakers have a slower reading speed in general, the reading durations in their respective first languages would need to be addressed in a follow-up study. Also, this would shed light on the influence of individual participants’ reading proficiency on our results (see below).

There are several possible explanations why, overall, our non-native speakers did not appear to produce pauses ‘with a foreign accent’. Pauses may be easier to acquire than other aspects of language because they are perceptually salient (Matzinger, Ritt, Fitch, in prep) and pausing is articulatorily easy, relative to phonemes or intonation patterns. Alternatively, pauses might not contribute to non-native speech production because the pause patterns in the speakers’ L1 and L2 might be similar and speakers simply transfer their L1 pause patterns to their L2. Such a transfer might imply that pauses result from very general cognitive mechanisms [53] and thus have a more universal character than other aspects of language. However, to substantiate this hypothesis, non-native English speakers of a typologically diverse set of languages, including many other native languages than German and Serbo-Croatian, would need to be tested in a larger-scale study. If such L2 speakers still pause with a native-like accent when speaking English, this would be evidence for a language universal character of pause patterns.

If pauses result from basic cognitive mechanisms, L2 speakers should also pause with a non-native accent in other second languages than English.

Although our study controls for many aspects necessary to investigate foreign speech production, there are some aspects that it does not address, but that could be addressed in potential follow-up experiments. Our study tested the role of pauses of highly proficient L2 speakers. Certainly, L2 proficiency might have an influence on the realization of pauses [8,34]. L2-typical pause patterns might be acquired rapidly (and should thus not contribute to non-native accents in any proficiency level), slowly but still more quickly than e.g. phonemes (and should thus only contribute to non-native accents in beginners), or not at all (and should thus contribute to non-native accents in any proficiency level). Therefore, testing L2 speakers of different proficiency levels in a follow-up experiment might reveal more about the cognitive constraints associated with speaking an L2, which might contribute to non-native speech production. Our present finding that pauses contribute little to the acoustic peculiarities of L2 production would be further corroborated if a comparable study with less proficient non-native speakers yielded similar results.

Furthermore, we caution that our relatively small sample size of 41 participants, although clearly adequate to reveal multiple statistically significant effects, might potentially be inadequate to reveal more subtle differences of smaller effect size. Thus, like any null result, our “no difference” findings should be viewed with some caution. However, the considerable time and effort required to derive, annotate and manually check pause data (more than 26,000 data points of which more than 2,700 were pauses comprise our current dataset) would remain a challenge for gathering much larger samples of participants.

We only included academically educated participants, from which a similarly high level of reading proficiency could be assumed. Testing people with a high reading proficiency might have contributed to the fact that, overall, we found similar pause patterns in native and non-native speakers. Highly proficient readers might be able to override challenges in foreign speech production when reading, but not when speaking freely (see below). Less proficient readers might not be able to mask difficulties in non-native speech production when reading, which might result in different pause patterns between native and non-native speakers. A potential follow-up study could explicitly test participants’ reading proficiency and include it as a predictor for pause patterns.

The text that the participants read out in our study contained punctuation marks. Punctuation marks are salient visual cues that might prime participants’ pause patterns [84]. We used a text containing punctuation marks to make the procedure as close to real-life reading situations as possible. How much punctuation contributes to adopting the pause patterns of an L2 in read speech could easily be tested in a follow-up study with texts without punctuation marks. Similar results using texts without punctuation marks could evaluate the possibility that the realization of pauses is determined by basic cognitive processes, because in such cases participants would not be primed by visual cues.

Results of our study on read texts might not be entirely transferrable to spontaneous speech, because pauses in reading aloud might also reflect reading difficulties or spelling-to-sound difficulties. In contrast, pauses in spontaneous speaking might reflect difficulties in conceptualizing the message or in linguistic formulation. Still, we argue that testing speakers during reading aloud has ecological relevance because it occurs in several real-life contexts, such as when reading aloud to children or in the (language) classroom. Especially during second language learning, reading aloud is a commonly practiced exercise [85]. Furthermore, reading is ideal for our purposes since it captures difficulties in articulation which are highly relevant for non-native speech production. Further, reading a complete story also captures pauses in longer stretches of speech, as opposed to reading or repeating isolated sentences (e.g. [8]). Thus, we feel that our paradigm adds a rigorous and useful new method to the existing literature on spontaneous speech.

One crucial point that our procedure cannot address is whether it is a transfer from L1 pause patterns that either hinders (because L1 and L2 pause patterns are different) or facilitates (because L1 and L2 pause patterns are similar) the acquisition of L2 typical pause patterns. Testing similarities or differences of different native languages’ pause patterns is almost impossible: even if speakers of different L1s are tested on similar tasks, such as reading out texts matched in syntax and content, translations can never be fully identical in syntactic structure or small nuances of content. Such minute differences might already shape pause patterns in a way that makes it difficult to determine these potential differences’ contribution to L2 accents. Nonetheless, testing the same speakers in their L1 and L2 would be useful to address the effect of individual speaking style on pause patterns [47].

### Conclusions and future work

We asked native and non-native speakers to read the same English text, thus excluding potential L1-specific morpho-syntactic factors from influencing the vocal output. We found that speakers inserted pauses into the reading stream in similar ways and at similar locations, and changed this pattern in similar ways at different reading tempi, regardless of their native language. The only difference between pause patterns in native and non-native speakers was that the non-native speakers made more pauses than the native speakers. This might reflect cognitive processing constraints in an L2 that result from other aspects of the L2 than pausing behavior per se. Overall, we conclude that in reading aloud, the influence of nativeness on the realization of pauses is marginal, suggesting that pauses play little role in the production of foreign accents in this context

## Supporting information

S1 Table Results of cross-linguistic studies suggesting that the numbers and durations of pauses in different languages are similar. (DOCX)

S2 Table Results of cross-linguistic studies suggesting that the numbers and durations of pauses in different languages are different. (DOCX)

S3 Table Results of studies on the numbers and durations of pauses during L2 speech. Results in the table concern comparisons between pauses in speakers’ L2s and these speakers’ L1s (as opposed to L1 speakers of the target L2).
(DOCX)

S4 Table Results of studies on the numbers and durations of pauses during L2 speech. Results in the table concern comparisons between pauses in speakers’ L2s and L1 speakers of the target L2.
(DOCX)

S5 Table Native language recognition ratings. (DOCX)

S6 Table Estimated variance components and standard deviations for the random intercept of participant of the full model exploring the effects of reading tempo, and nativeness on the total reading time. (DOCX)

S7 Table Estimated variance components and standard deviations for the random intercept of participant of the full model exploring the effects of reading tempo and nativeness on pause-to-utterance ratio. (DOCX)

S8 Table Estimated variance components and standard deviations for the random intercept of participant of the full model exploring the effects of reading tempo, and nativeness on the duration of individual pauses. (DOCX)

S9 Table Estimated variance components and standard deviations for the random intercept of participant of the full model exploring the effects of reading tempo, nativeness and in-text position on the occurrence frequency of pauses. (DOCX)

S1 Appendix Post-experiment questionnaire. (DOCX)

S2 Appendix Annotated text *The boy who cried wolf*. (DOCX)
