# Supplementary material for: Non-native speaker pause patterns closely correspond to those of native speakers at different speech rates
Source: PLoS One. 2020 Apr 3;15(4):e0230710. doi: 10.1371/journal.pone.0230710 (PMC7124187; doi:10.1371/journal.pone.0230710)
Supplement: S1 Table — (DOCX) [file pone.0230710.s001.docx]

**S1 Table. Results of cross-linguistic studies suggesting that the numbers and durations of pauses in different languages are similar.**

| **Characteristics considered** | **Results** | **References** |
| --- | --- | --- |
| **Number of pauses** | English ≈ French ≈ Spanish | (1) |
|  | French ≈ English | (2) |
|  | English ≈ Croatian | (3) |
|  | adjustments to different speech tempi: Dutch ≈ English ≈ French ≈ Italian ≈ Romanian ≈ Spanish | (4) |
| **Duration of pauses** | English ≈ French ≈ Spanish | (1) |
|  | English ≈ Croatian | (3) |
|  | English ≈ Chinese | (5) |
|  | durational adjustments to different speech tempi: Dutch ≈ English ≈ French ≈ Italian ≈ Romanian ≈ Spanish | (4) |
