# Supplementary material for: Non-native speaker pause patterns closely correspond to those of native speakers at different speech rates
Source: PLoS One. 2020 Apr 3;15(4):e0230710. doi: 10.1371/journal.pone.0230710 (PMC7124187; doi:10.1371/journal.pone.0230710)
Supplement: S2 Table — (DOCX) [file pone.0230710.s002.docx]

**S2 Table. Results of cross-linguistic studies suggesting that the numbers and durations of pauses in different languages are different.**

| **Characteristics considered** | **Results** | **References** |
| --- | --- | --- |
| **Number of pauses** | English > French | (6,7) |
|  | Spanish > English | (8) |
|  | English > Turkish | (9) |
|  | French > German | (10) |
| **Duration of pauses** | French > English | (2,6) |
|  | Russian > English | (11) |
|  | French > German | (10) |
|  | Spanish > English ≈ French ≈ German > Italian | (12) |
|  | durational change in older age: German > French | (13) |
