# Supplementary material for: Non-native speaker pause patterns closely correspond to those of native speakers at different speech rates
Source: PLoS One. 2020 Apr 3;15(4):e0230710. doi: 10.1371/journal.pone.0230710 (PMC7124187; doi:10.1371/journal.pone.0230710)
Supplement: S3 Table — Results in the table concern comparisons between pauses in speakers’ L2s and these speakers’ L1s (as opposed to L1 speakers of the target L2). (DOCX) [file pone.0230710.s003.docx]

**S3 Table. Results of studies on the numbers and durations of pauses during L2 speech.** Results in the table concern comparisons between pauses in speakers’ L2s and these speakers’ L1s (as opposed to L1 speakers of the target L2).

| **Characteristics considered** | **Results** | **L1** | **L2** | **References** |
| --- | --- | --- | --- | --- |
| **Number of pauses/Pause rate** | L2 = L1 | Russian, Ukrainian, Mandarin | English | (14) |
|  |  | Japanese | English | (15) |
|  | L2 > L1 | Russian | English (intermediate and high proficiency) | (11) |
|  |  | Thai | English | (16) |
|  |  | French | English | (17) |
|  |  | German | French | (2,10) |
|  |  | French | German | (2,10) |
|  |  | English, Turkish | Dutch | (9) |
| Number of pauses between utterances | L2 = L1 | English, Turkish | Dutch | (18) |
| Number of pauses within utterances | L2 > L1 | English, Turkish | Dutch | (18) |
|  |  | German | French | (10) |
|  |  | French | German | (10) |
|  |  | “a range of different L1 backgrounds” | English | (19) |
| **Duration of pauses** | L2 = L1 | Russian, Ukrainian, Mandarin | English | (14) |
|  |  | Japanese | English | (15) |
|  |  | Russian | English (intermediate proficiency) | (11) |
|  |  | French | English | (17) |
|  | L2 > L1 | German | French | (2) |
|  |  | Turkish, English | Dutch | (18) |
|  |  | German | English, French | (20) |
|  |  | English, Turkish | Dutch | (9) |
|  | L2 < L1 | German | French | (10) |
|  |  | French | German | (2,10) |
|  |  | English | Russian (high proficiency) | (11) |
| Duration of pauses between utterances | L2 = L1 | English, Turkish | Dutch | (18) |
| Duration of pauses within utterances | L2 > L1 | English, Turkish | Dutch | (18) |
