# Supplementary material for: Non-native speaker pause patterns closely correspond to those of native speakers at different speech rates
Source: PLoS One. 2020 Apr 3;15(4):e0230710. doi: 10.1371/journal.pone.0230710 (PMC7124187; doi:10.1371/journal.pone.0230710)
Supplement: S4 Table — Results in the table concern comparisons between pauses in speakers’ L2s and L1 speakers of the target L2. (DOCX) [file pone.0230710.s004.docx]

**S4 Table. Results of studies on the numbers and durations of pauses during L2 speech.** Results in the table concern comparisons between pauses in speakers’ L2s and L1 speakers of the target L2.

| **Characteristics considered** | **Results** | **L1** | **L2** | **References** |
| --- | --- | --- | --- | --- |
| **Number of pauses/Pause rate** | L2 = L1 | Russian | English (high proficiency) | (11) |
|  | L2 > L1 | Russian | English (intermediate proficiency) | (11) |
|  |  | Korean | English | (21) |
| **Duration of pauses** | L2 = L1 | Russian | English (high proficiency) | (11) |
|  | L2 < L1 | Russian | English (intermediate proficiency) | (11) |
