# Supplementary material for: Non-native speaker pause patterns closely correspond to those of native speakers at different speech rates
Source: PLoS One. 2020 Apr 3;15(4):e0230710. doi: 10.1371/journal.pone.0230710 (PMC7124187; doi:10.1371/journal.pone.0230710)

**S5 Table. Results of a native language recognition test for our pool of speech samples of English, German and Serbo-Croatian native speakers (S1-S41) reading an English text.** T = native English raters identified the speakers’ nativeness status correctly. F (gray) = native English raters misclassified the speakers’ nativeness status. Four German native speakers were misclassified as being English native speakers by one or two of the raters each, but correctly identified as being non- native by the other raters. Four English native speakers were misclassified as being non-native speakers of English by one or two of the raters each, but correctly identified as native speakers by the other raters.


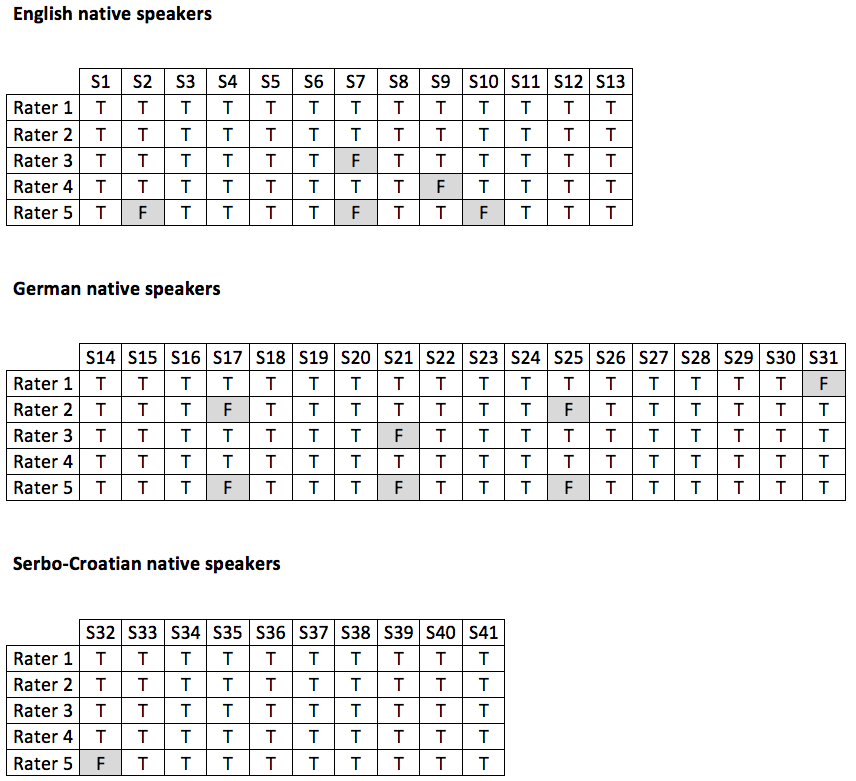

Supplement: S5 Table — (DOCX) [file pone.0230710.s005.docx]
