# Supplementary material for: Non-native speaker pause patterns closely correspond to those of native speakers at different speech rates
Source: PLoS One. 2020 Apr 3;15(4):e0230710. doi: 10.1371/journal.pone.0230710 (PMC7124187; doi:10.1371/journal.pone.0230710)
Supplement: S8 Table — (DOCX) [file pone.0230710.s008.docx]

**S8 Table. Estimated variance components and standard deviations for the random intercept of participant of the full model exploring the effects of reading tempo, and nativeness on the duration of individual pauses.**

| **Random effect** | **Term** | **Variance** | **Standard deviation** |
| --- | --- | --- | --- |
| **Participant** | Intercept | 0.04 | 0.20 |
| **Residual** |  | 0.03 | 0.17 |
