# Supplementary material for: Non-native speaker pause patterns closely correspond to those of native speakers at different speech rates
Source: PLoS One. 2020 Apr 3;15(4):e0230710. doi: 10.1371/journal.pone.0230710 (PMC7124187; doi:10.1371/journal.pone.0230710)
Supplement: S1 Appendix — (DOCX) [file pone.0230710.s010.docx]

# S1 Appendix. Post-experiment questionnaire.

**Questionnaire for participants**

| ID: |  |  |  |
| --- | --- | --- | --- |
| Age: |  | Date: |  |
| Gender: |  | Nationality: |  |

**PLEASE ANSWER THE FOLLOWING QUESTIONS:**

**Part I: Your language(s)**

1) What is your first language (please include not only the standard but also non-standard variety (i.e. your “dialect”)?

2) Did you grow up bilingually? If so, what are your first languages?

3) Which other languages do you speak (please specify estimated proficiency level)?

**Part II: Taking a closer look at your English (only for participants whose first language is not English)**

1) At what age did you start learning English?

2) Which variety of English do you aim to speak?

3) Have you ever taken English pronunciation classes? Please specify (For which variety? For how long? etc.)

4) Do you aspire to have native-like pronunciation?

5) Which other influences might have shaped the way you speak English (i.e. for example having lived in an English-speaking country for a period of time, media exposure, etc.)?
