# Supplementary material for: Non-native speaker pause patterns closely correspond to those of native speakers at different speech rates
Source: PLoS One. 2020 Apr 3;15(4):e0230710. doi: 10.1371/journal.pone.0230710 (PMC7124187; doi:10.1371/journal.pone.0230710)
Supplement: S2 Appendix — (DOCX) [file pone.0230710.s011.docx]

# S2 Appendix. Annotated text *The boy who cried wolf.*

For determining the positions of pauses, we used the following classification:

[MA] pauses occurring at punctuation marks
[UM] pauses occurring at unmarked clause or phrase boundaries

Pauses at other positions in the text (e.g. between “There” and “was”, or between “was” and “once”), were classified as “other” and are not indicated in the text below. In total, 76.29 % of all pauses made occurred at punctuation marks, 15.13 % occurred at unmarked clause or phrase boundaries, and 8.58 % occurred at other positions in the text.

**The Boy who Cried Wolf**

There was once a poor shepherd boy **[UM]** who used to watch his flocks **[UM]** in the fields **[UM]** next to a dark forest **[UM]** near the foot of a mountain. **[MA]** One hot afternoon, **[MA]** he thought up a good plan **[UM]** to get some company for himself **[UM]** and also have a little fun. **[MA]** Raising his fist in the air, **[MA]** he ran down to the village **[UM]** shouting ‘ **[MA]** Wolf, **[MA]** Wolf.’ **[MA]** As soon as they heard him, **[MA]** the villagers all rushed from their homes, **[MA]** full of concern for his safety, **[MA]** and two of his cousins even stayed with him for a short while. **[MA]** This gave the boy so much pleasure **[UM]** that a few days later **[UM]** he tried exactly the same trick again, **[MA]** and once more he was successful. **[MA]** However, **[MA]** not long after, **[MA]** a wolf **[UM]** that had just escaped from the zoo **[UM]** was looking for a change **[UM]** from its usual diet **[UM]** of chicken and duck. **[MA]** So, **[MA]** overcoming its fear of being shot, **[MA]** it actually did come out from the forest **[UM]** and began to threaten the sheep. **[MA]** Racing down to the village, **[MA]** the boy of course cried out even louder **[UM]** than before. **[MA]** Unfortunately, **[MA]** as all the villagers were convinced **[UM]** that he was trying to fool them a third time, **[MA]** they told him, ‘ **[MA]** Go away **[UM]** and don’t bother us again.’ **[MA]** And so the wolf had a feast.
